# Determinants of Sedentary Behavior, Motivation, Barriers and Strategies to Reduce Sitting Time in Older Women: A Qualitative Investigation

**DOI:** 10.3390/ijerph110100773

**Published:** 2014-01-07

**Authors:** Sebastien F. M. Chastin, Nicole Fitzpatrick, Michelle Andrews, Natalie DiCroce

**Affiliations:** Institute of Applied Health Research, School of Health and Life Science, Glasgow Caledonian University, Cowcaddens Road, Glasgow G4 0BA, Scotland, UK; E-Mails: nfitzpatrick1986@gmail.com (N.F.); sandrews@gcal.ac.uk (M.A.); ndicrocw@gcal.ac.uk (N.D.)

**Keywords:** sitting, frailty, ageism, physical activity, intervention

## Abstract

Sedentary behavior defined as time spent non-exercising seated or reclining posture has been identified has a health risk and associated with frailty and disablement for older adults. Older adults are the most sedentary segment of society. To date no study has investigated the determinants of sedentary behavior in older adults. This study reports a qualitative investigation of the determinants of sedentary behavior, strategies and motivator to reduce sitting time by structured interviews in a group of community dwelling older women (*N* = 11, age 65 and over). Older women expressed the view that their sedentary behavior is mostly determined by pain which acts both as an incentive to sit and a motivator to stand up, lack of energy in the afternoon, pressure from direct social circle to sit and rest, societal and environmental typecasting that older adult are meant to sit, lack of environmental facilities to allow activity pacing. This qualitative investigation highlighted some factors that older adults consider determinants of their sedentary behavior. Some are identical to those affecting physical activity (self-efficacy, functional limitations, ageist stereotyping) but some appear specific to sedentary behavior (locus of control, pain) and should be further investigated and considered during intervention design. Tailored interventions that pay attention to the pattern of sedentary behavior of individuals appear to be supported by the views of older women on their sedentary behavior.

## 1. Introduction

Older adults aged 65 and over are the fast growing segment of the World’s population. According to the World Health Organization, the number of older adults are likely to double by 2050 [1]. Within this time period the global population of over 80 year olds is forecasted to quadruple to nearly 400 million and in the UK alone, 85 year olds and over are the fastest growing population group [2]. In addition, the age-related public expenditure in the UK is projected to increase to 26% of GDP by 2057. 

A large part to this age related socio-economic burden is due to the societal and individual cost of managing multiple chronic disease and disablement in later life. Furthermore, there is strong evidence that physical activity is a modifiable health behavior that can prevent chronic conditions, help maintain independence and increase the quality of life and wellbeing in later life. 

However, current epidemiological data shows that older adults are also the most sedentary segment of the population. In the UK and USA, older adults spend on average 70% of their waking hours being sedentary and at least half of everyone over 70 years old sits for 80% of the day [3,4]. In the UK, the healthcare cost of inactivity is estimated to be £8.5 billion per year, which equates to 10% of the national health care budget [5]. 

Sedentary behavior is defined as time spent in non-exercising, seated or reclining pursuits, such as watching television, sitting in motorized transport or in front of a computer at work [6]. Recent evidence from multiple lines of enquiry show that sedentary behavior has deleterious effects on health and is associated with an increased risk of chronic disease, disablement and premature death in older adults [3,7,8,9,10]. Furthermore, a recent study also found that older adults who are sedentary are less likely to age successfully in the physical, psychological and sociological domain [11]. The effect of sedentary behavior on health is two-fold [12]. Firstly, the amount of time spent in sedentary pursuits tends to displace the time spent being active and therefore prevents individuals from reaping the health benefits that physical activity brings. More recently it has also been shown that sedentary behavior has a specifically deleterious effect on a person’s health, independent of the amount of physical activity that is carried out [12]. Indeed, it is possible to carry out the recommended 30 min of daily physical activity and yet spend the rest of the day sitting down.

Consequently, several countries (USA, Canada and the UK) and the World Health Organization have issued guidelines for older adults to limit the amount of time spent sitting and recommended that research is carried out on interventions which reduce the amount of time that older adults spend sitting [13,14,15]. 

To date only one study has attempted to modify the sedentary behavior in older adults [16]. It showed that it is possible to change the amount of sitting time in young and healthy older adults using tailored behavioral interventions, but there is no evidence to show that this approach would transfer to older and frailer individuals. In addition the type of behavioral interventions that were addressed by this study only focus on personal factors and entirely neglects the interpersonal, environmental and societal factors that might affect the amount of time which older adults spend sitting down in any given day.

Current theoretical frameworks [17,18] hypothesize that a complex interplay between personal circumstances, environmental and social factors determine sedentary behavior. Research on the determinants of physical activity among older adults has shown that environmental social, behavioral and cognitive factors are key for the initiation, and long-term maintenance, of physical activity [19,20]. However, it is not known if these same factors also determine sedentary behavior. 

In fact, the current dearth of information about the determinants of sedentary behavior is the single most important factor in limiting the development of interventions which modify sedentary behavior in older adults. While some very limited cross-sectional quantitative information exists in this area [17], no studies have attempted to gather the views and opinions of older adults into the factors which determine their sedentary behavior. The aim of this study was to address this gap in knowledge by gathering this information from community-dwelling older adults with various levels of independence and frailty.

## 2. Experimental Section

### 2.1. Study Design

A qualitative research design in the form of semi-structured interviews was used in this study. One of the major advantages of the semi-structured interview technique is its adaptability. The interviewer can use what the individual is saying to modify the interview with follow up ideas and probing questions in order to thoroughly investigate an issue [21]. The semi-structured interviews conducted in this study were based on a simple set of questions which probed the participants on the following areas:

(1) The reason they sit.
(2) The reason they stop sitting and stand up.
(3) Any simple daily life strategy they might adopt to sit less.

Experts in gerontology reviewed the questions to ensure that they were adequate and that the language was appropriate for this audience.

### 2.2. Ethics

The study was approved by Glasgow Caledonian University, School of Health and Life Science ethics’ committee and Glasgow City Council lunch clubs’ management. All participants were given written and verbal descriptions of the study and signed a consent form. They were also given the option to opt out of the study at anytime. 

### 2.3. Sample

A sample of convenience comprising 11 women took part in this study. Participants were recruited from Glasgow City Council lunch clubs. For participants to be included in the study they had to be community-dwelling woman aged 65 or over, living independently in their own home and capable of speaking and understanding English. 

### 2.4. Participant Profile

Prior to interview participants were asked for some socio-demographic information including; age, the type of accommodation they lived in, and with whom, and to what level they had been educated. Their socio-economic status was estimated using the Scottish Multiple Index of Deprivation. The participants also filled a short battery of questionnaires designed to estimate the amount of time they spent being sedentary (Sedentary Behavior Questionnaire, SBQ [22]), their self-reported physical and mental health status (Short Form Health Survey, SF12 [23]) and their level of social support (Functional Social Support Questionnaire, FSSQ [24]). In addition their psychological orientation towards life and ageing were assessed using both the Selector, Optimisor, Compensator (SOC) life management questionnaire [25] and sense of coherence questionnaire [26].

### 2.5. Setting

The study took place in three Glasgow City Council lunch clubs for older adults as they are social spaces were older adults gather twice a week for lunch and to participate in social activities. The majority of older adults are driven to and from the lunch clubs in community buses and while one lunch club was located in the highest social economic area of Glasgow city centre, two were located in low social economic suburban areas of the city. 

All the interviews took place in rooms that were separated from the lunch hall and three researchers were present during each interview; an interviewer, a note taker who took detailed minutes of the discussion and a researcher who wrote down ideas on flipcharts as recommended by [27].The flipchart was used to record all the ideas and information given by the participants and the notes served as a point of reference during analysis. At the end of the interview the participants were asked to review these materials in order to verify that they were a truthful account of what had taken place, the discussion and their opinion.

### 2.6. Analysis

O’Connel *et al.*, [28] recommended that consideration should be given to the transcription of data, arguing that the recording and transcription of data was only necessary if they would help generate useful extra information above that which could be obtained anyway. As the main focus of this study was a primary identification of the determinants of the sedentary behavior of participants, the interviews were neither audio-recorded nor transcribed. A framework analysis [29] was adapted, using the method developed by the UK Centre for Social Research for data management. It involved using the flipchart and notes at the same time as the interview was being conducted. This qualitative method was used to classify the information given by the individual participants into themes and sub-themes. At the end of the interview the themes and sub-themes were shared with the participants to ascertain if they were a true reflection of their opinions and the analysis and classification were cross-checked by the three researchers who were present. Data from all participants was then collated and examined for patterns and associations using an inductive thematic analysis approach [30].

## 3. Results

### 3.1. Sample

Two participants decided to opt out of the study after the interview. The results presented here are therefore based upon *N* = 9. The socio-economic and questionnaire data of the participants are given in Table 1. 

### 3.2. Findings

Framework analysis was used to organize the data into three categories of information; sedentary behavior, upright behavior and strategies which participants use to reduce their sitting time. The sub-themes in each of these categories, and the data which supports them, are presented in Table 2 and Table 3. 

#### 3.2.1. Sedentary Behavior

In the “sedentary behavior” theme there was a lot of similarity in the participants’ answers across all sub-themes. Participants appear to share the same sitting patterns throughout the day. Most participants reported that they either sat more during the afternoon or all day. This appeared to be a common way for participants to manage their energy levels throughout the day. The activities they performed while sitting were also consistent and were generally solitary activities, such as television watching or cognitively demanding tasks such as doing crosswords. Participants’ answers also included group activities such as bingo which are common activities to take place during the lunch clubs.

Reasons that people gave for sitting seem to fall into five main themes: physical complaints, lack of environmental facilities and stimuli, peer and societal pressure, pleasure and relaxation and mental health reasons. Physical complaints appeared to be the main personal reasons that participants would sit. Pain felt in the standing position, fatigue experienced while standing and functional limitations which make standing difficult were reported by almost all participants as being the most important reason for sitting down. 

All participants complained about the environment they live in, claiming that it does not offer adequate stimuli to encourage them to stand up or enough facilities to allow them to be active. In particular they reported that the lack of resting places outside the home strongly limited their motivation or confidence to be active. Most participants said they would walk more if they could find resting places at staggered intervals in public spaces. Instead they chose to sit down indoors because they feared being too tired or embarrassed if they walked outside and were ‘caught short’. In addition they felt that activities and facilities targeted to older adults involved sitting down for long periods. In fact, they felt that activities provided for older adults were mostly, if not always, designed to be undertaken in a seated posture. For example, all the lunch club activities, such as bingo, were based around sitting. This later theme overlaps with the common view the participants had that society, friends and family expect older adults to sit as their main mode of living. They all felt typecast as “not useful” or “unable”, particularly under the label of “pensioners”. For example, they related that family members would commonly expect them to sit and would prevent them from being active. While they recognized that this was a caring gesture, they all expressed the opinion that this took opportunities for being active and independent away from them. 

At the same time, they expressed confidence issues about being upright independently and how this limited their motivation for standing up through a fear of falling, for example, or fear of being a burden. In addition to lack of confidence, lack of motivation caused by depression was another mental reason participants gave for why they would sit for so long. In contrast, participants also viewed sitting as a well earned right to relax but only if it is a free choice and devoid of guilt. 

#### 3.2.2. Upright Behavior

There were a lot of similarities between the participants’ answers in this theme. The pattern of time spent upright was the inverse to the sitting pattern with the morning being the most likely time of the day that participants reported standing more often. The main activities which the participants reported carrying out during this time were household chores or activities of daily living and walking. Making a cup of tea was also reported as being a very common activity. Reasons to stand up fell into six main categories; relieving physical discomfort, boredom and depression, personal characteristics (whether they are energetic people) and habitus, sense of worth, social and leisure activities and environmental factors. 

Standing up regularly was reported as a necessity by almost all the participants for relieving pain and stiffness experienced after sitting for too long. In addition to relieving physical symptoms, participants also reported that they stand up to fight depression and boredom. Some expressed the view that they fabricate reasons to stand as a coping mechanism against depression and boredom which sets in after sitting for too long. The ability to stand and engage in activities was reported as an important demonstration of independence and self-worth. Being useful, self caring and caring for others were seen as strong motivations to interrupt sitting. A lot of participants felt that upright behavior is down to personal characteristics and motivations. These appear to arise from habitus, such as a lifetime spent looking after a busy household, an active lifestyle or bad experience of being immobilized at some point in the past. However, most participants acknowledged the need for external stimuli coming from the community, social circle and family as a strong determinant for being upright. Finally, a safe environment that allows for rest was also a key factor given by the participants for motivating them to get and stay upright.

#### 3.2.3. Opinions about Decreasing Sitting Time

Opinions about reducing sitting time differed widely, ranging from a clear refusal to a clear will and interest in sitting less. In between these two extremes, participants also expressed the view that they could not see the benefits of standing more as they felt entitled to sit at their age or that standing up more would interfere with the strategies they had put in place to make their lives easier to manage. Finally some participants reported that although they could see and understand the benefits, standing was not something they could engage with because of their physical or mental state. 

**Table 1:** Participants demographics and questionnaire scores (SBQ: Sedentary Behavior Questionnaire, FSSQ: Functional Social Support Questionnaire, SF-12_PCS: Physical health, SF-12_MSC: Mental health).

Participants	Age	SIMD ^a^	Accommodation	Marital Status	Education Level Reached	Sedentary Behavior ^b^ (h/day)	FSSQ ^c^	SF-12_PCS ^d^	SF-12_MCS ^d^	SOC ^e^	Coherence ^f^
1	70	3	Flat	Widowed	Secondary	7	4.25	20.8	32.6	LBS	56
2	70	3	Flat	Widowed	Primary	>8.5	5.00	23.7	46.7	C	67
4	82	2	Flat	Married	Secondary	>7	4.25	53.2	57.3	S	70
5	77	2	Flat	Divorced	Secondary	>8.5	4.00	28.3	31.8	S	62
6	82	2	Flat	Widowed	Secondary	14	4.75	46.3	57.1	O	84
7	87	2	House	Married	Primary	8.5	4.5	52.6	55.5	C	81
9	92	3	House	Widowed	Secondary	5	4.25	28.7	62.5	O	79
10	83	1	House	Widowed	Secondary	3	5.00	35.2	56.9	C	77
11	72	1	Flat	Married	Higher Education	>6	3.38	37.5	20.7	LBS	53

^a^ Socio-economic status scale from 1 (low) to 5 (high); ^b^ Self reported sedentary time (SBQ); ^c^ Scale from 1 (low perceived support) to 5 (high perceived support); ^d^ For the SF12 Physical Component Score (PCS) and Mental Component Score (MDS), a score of 50 is the norm. A score > 50 represent better than the norm self reported health; ^e^ Life management questionnaire: S = Selector, LBS = Loss based selection, C = Compensator, O = Optimisor; ^f^ 13 item Sense of coherence questionnaire: Higher scores represents a higher sense of coherence.

**Table 2:** Sedentary behavior themes.

Participant	Time of Day	Primary Reason to Sit	Secondary Reason to Sit	Activities in Sitting
1	Afternoon (Peace of mind after stressful morning)	Pain (arthritis)	– Family and friend pressure to rest and who take over most task taking away opportunities to stand up (labelled as “pensioner”). – Fear of walking outside because not enough places to rest (in sitting), fear of embarrassment if could not manage without rest. – Relaxation (with distracting activities; must be guilt free). – Fatigue: organise day so that she can feel guilt free having done everything in morning. – Poor sleep night before. – Enjoy sitting (only if guilt free).	Crosswords
2	Afternoon, depends on effect of medications	Pain-arthritis	– Family, friend and others expect her to sit and be dependent. – Fear of walking outside because not enough places to rest (in sitting), fear of embarrassment if could not manage without rest. – Relaxation (with distracting activities; must be guilt free). – Fatigue: Tries to make things as easy and fatigue free as possible, do all necessary activities first in the morning when feel less fatigued. – Likes routine and sitting is part of the routine.	Crosswords
4	Afternoon	Fatigue—take frequent break from activity	– Lacks motivation to be active outside of daily shore. – Feels there is nothing to do for people her age outside of family and lunch club. – All community activities are in sitting and boring. – Medical (back pain, feel must sit because of hip replacement). – Family encourage her to sit and take over chores and self care. – Sit is what old people do. – View sitting as reward she is entitled to. – Sit because do not want to burden other in case she needs help.	Watching TV, Knitting
5	Most of day	Pain-arthritis Least pain experienced in seated posture	– Depression. – Lack of motivation to do anything.	Watching TV
6	As little as possible	Relaxation (always on the move, and busy)	– Loss of confidence to do things.	Crossword Watching TV
7	Afternoon (2 h) Evening (4 h)	Fatigue—feel everything demands more energy because of arthritis	– More interesting activities in sitting . – Nothing to do family and health care system take away chores. – All proposed activities in the community are in sitting. – Fear of falling. – Knees are not good.	Watching TVBingo
9	Afternoon	Feel forced to sit because seen as a person who must sit and encouraged to do so. Not enough opportunity and activities. All organised activities such as lunch club are seated.	– Limited physical ability. – Pain.	Singing (now required to sit while performing this activity in social events), Bingo
10	Afternoon	Mobility issues	– Poor weather must stay indoors. – Fatigue after 20 minutes of walking. – Social and family pressure.	Watching TV
11	Most of the day	Necessary because of mobility issues	– Limited to activities in standing that require only one hand. – Social pressure from family, home help to do as little as possible.	Moves to music, Puzzles

**Table 3:** Upright behavior themes.

Participant	Time of Day	Primary Reason to be Upright	Secondary Reason	Activity in Standing
1	Morning, like doing things that need to be done first	Relieve pain and stiffness	– Habit (had 8 kids always been busy) – Show independence and sense of purpose in taking part in activities – Depressed if sit too long – Prefer to stand feel less guilty	House chores Making a cup of tea Create things to do
2	Morning	Relieve pain and stiffness from sitting too long	– Sense of purpose and independence – To not just be the old person in the chair. – Way of managing depression	House chores Making a cup of tea
4	Morning	Feeling that there is always something to be done in the house	– Good weather (rare) – Bedroom up the stairs – Being in an environment that allow for frequent short periods of sitting as get tired after 20 minutes of walking or standing – Relieve stiffness and pain. – Relieve boredom – Family commitments	House chores Gardening Shopping with daughter
5	Minimal time spent standing	Relieve boredom Do not enjoy sitting	– Use standing activities as a coping mechanics to relieve depression	House chore Making a cup of tea
6	As much as possible throughout day	Self motivation, knows exercising and moving is good. Bad personal experience in hospital being immobilised for too long	– Relieve boredom – Going outdoors – Socialising	Walking Making a cup of tea Shopping Taking care of others
7	Minimal time spent standing	Relieve pain and stiffness	– Good weather – Enjoyment of caring for others – Dancing – If action of standing is pain free	Walking around the house Caring for others Dancing
9	As much as possible throughout day	Self motivation and determination	– Does not enjoy sitting (already sit more than she would like) – Has always taken care of other – Active background – Relieve pain and stiffness – Feel capable of self caring – Managing stiffness	Caring for other people Calling numbers at bingo
10	Morning	Relieve pain and stiffness from sitting too long	– Feel more energised – Motivation from family and social group	Walking 20-30 before too fatigued Structured exercise in social context
11	Morning	Self motivation determination	– Relieve pain and stiffness from sitting too long – Therapeutic exercises – Use of walking aid – Mobility around house (safe) – Music	Self directed exercises Minimal walking around the house

#### 3.2.4. Strategies Thought to Decrease Sitting Time

Despite differing views on the benefits of reducing the amount of time participants spent sitting, they offered opinions about how this could be best achieved. Their answers fell into six themes; the provision of community and social opportunities, the safety of their environment and transportation, motivation, tailored activities and caring for others. All participants said that they felt more social and community-based opportunities to be active would help them reduce their sitting time. In particular they reported that community and social activities should not revolve solely around sitting but instead should offer more enjoyable alternatives such as gardening, dancing, listening to music or shopping. 

They expressed the opinion that it was vital to have access to a safe environment where they could be active, yet rest when they needed. They felt that transportation and access to facilities, such as community-based activities was crucial. Caring for others and feeling useful was commonly reported as being a strategy that they would both enjoy and that would be a strong motivator to change their sedentary behavior. The majority of participants also felt that reducing sitting time is a matter of personal motivation. However support from close a social or family circle and peers was also viewed as an important motivating factor for initiating and maintaining a change in behavior. 

However, there were differing views about the best time of the day to promote a change in behavior around sitting. Some felt that the morning is the most suitable time as participants have more energy while others thought that they stood a lot in the morning and would need help to be more active in the afternoon. Most participants said that a good strategy for promoting behavioral change over sitting less and standing up more would involve thinking of more simple and short activities that engaged them on a regular basis, particularly at home. For example, making tea more often, was the most commonly reported activity which fell into this category. 

## 4. Discussion

Sedentary behavior, or time spent doing non-exercising, reclining and seated activities, is emerging as an important public health issue, particularly amongst older people who are the most sedentary group within the population. Several national guidelines recommend a reduction in sedentary behavior for the older adult population but currently there is little knowledge about what determines sedentary behavior within this population group and how to develop interventions which leads them to change their behavior [13]. This is the first study to examine older people's views and perceptions about what determines their daily sedentary behavior. The findings of this study help identify opportunities for intervention and barriers to promoting sedentary behavior change.

Sedentary behavior appears to be viewed by older women as a necessity. Sitting time is built into their daily routines as a way of managing chronic disease symptoms, such as pain and stiffness, renewing or conserving energy levels and making life easier and more enjoyable. During these periods, older women do not see sitting as an unhealthy behavior but rather as a positive coping strategy which enables them to remain functional and independent. A number of participants actually challenged the view that sedentary behavior is intrinsically unhealthy and should be reduced as they felt “entitled” to sit whenever they wanted or needed to. In addition, sedentary time is often centered on activities that have either a social nature or provide mental stimulation such as: playing bingo, reading or doing the crossword. Older women deemed these activities, which are all performed sitting down, to be positive, pleasurable and beneficial to their wellbeing. 

However, all the participants interviewed recognized that sitting too much could not be a healthy thing to do. However, this did not stem from an awareness of the detrimental effects of sedentary behavior on health. Instead, it was a result of personally experiencing the short-term consequences of sitting for prolonged periods, which most commonly included increased pain, stiffness and a depressive mood. Outside of the sedentary periods which they deemed necessary, periods of sedentary time were generally viewed as a bore and unpleasant experience. 

Sitting too much also appears to be socially undesirable for older women and is strongly linked to ageism [31]. Concerns about being judged as “lazy” or “not useful” seemed to create difficulties in acknowledging to themselves and others how long they genuinely sat down for. All participants felt that there was a social stigma attached with sedentary behavior for older people and that society expects them to sit all day. It is interesting to note that some recent epidemiological studies found that both older men and women self-report lower sitting time than younger age groups [32]. This is in complete contrast to objective reports [4] and hints that self report measures are strongly biased by social desirability. The feeling that there is a social stigma attached with sedentary behavior might be very pervasive amongst older adults, leading them to under-reporting of sitting time.

There is a sense amongst older women, that they are encouraged or even forced, to sit more than they wished to by the activities available to them and the social, community and urban environments they experience. This extends to the attitude of their family, friends and carers who commonly encourage older women to sit and actively discourage physical activity. While the participants acknowledged that these might be benevolent gestures, they felt strongly that it typecasts older adults as inherently dependent and removes their sense of purpose. There was a strong and unanimous desire to challenge this perception. 

From the data, it was possible to identify some perceived determinants of sedentary behavior which fit within the personal, inter-personal and environmental categories of the current ecological models of sedentary behavior [17,18].

In the personal category the most common perceived determinant of sitting appear to be symptoms of chronic diseases such as pain, stiffness and fatigue. In particular, pain and stiffness seem to act both as reason to sit and a reason for breaking periods of sedentary behavior. While the sample of this study might not be representative of the general older adult population, their symptoms are very representative of this group [33,34]. It is therefore conceivable that pain and stiffness play an important role in determining sedentary behavior amongst older women. In addition, the data suggests that these, and in particular fatigue, might directly affect the behavior patterns of sitting. 

In this study, most participants reported arthritis-related stiffness and pain. Therefore they should be more likely to experience difficulties in the morning [35] and as result spend more time sitting. [36]. However, surprisingly, the temporal pattern of behavior amongst all participants in this study appears to be the complete opposite of this. Everyone interviewed reported that they organize their day so that they can do what they have to in the morning and rest in the afternoon. This pattern is similar to what has been recently observed using objective measures of physical activity and sedentary behavior [37] and is consistent with reports of patterns of daytime rest and napping in older adults [38]. 

This pattern could be interpreted as an autonomic habitus [39] that has been developed to manage fatigue and energy expenditure. However, it is not clear whether this is a result of a perceived physiological fatigue or an innate way of regulating energy expenditure. Consequently fatigue might be a direct determinant of sitting especially in the afternoon or it might be fatigue avoidance. Or it could simply be that older women feel more capable in the morning, as reported by some participants in this study. Regardless of the underlying reason for this pattern, the pattern itself should be acknowledged by any interventions which aim to decrease the amount of time spent sitting.

Mobility issues were also reported as being primary reason for sitting. Some of these reasons were physical limitations and impairments directly affecting an individual’s ability to stand and remain in upright postures. While other reasons seem more likely to be related to low self-efficacy [40]. Some participants said that they sit a lot because they are scared of being active and suddenly finding they are tired and unable to cope. These well known determinants of physical activity in older adults [41] seem to also affect sedentary behavior. 

Depression is another known personal correlate of sedentary behavior [7] that some of the participants gave as a reason for remaining sedentary. They explained that depression affected their motivation to stand up and be active. 

Within the interpersonal category, the same ageist stereotypes and processes [42] that affect the promotion of physical activity in older adults [43] also seem to encourage sedentary behavior in this group. Although they may have good intentions, friends, family and carers may be overbearing in their desire to look after older adults, with participants reporting that they felt “molly-coddled” or “treated with kid gloves”. The most commonly reported activities that participants did while standing were in accordance with the social norms and tasks of daily living: house chores and taking care of others. Removing these social norms and providing greater support to older women than is needed reduces the opportunity for them to stand up and feel independent on a daily basis, in turn affecting their self-efficacy. Some of the participants actually said that they limited their standing activities out of a fear of being a burden to relatives or carers, in case they fell, got fatigued and did not cope while upright. 

The lack of provision of community-based activities, facilities and services which encourage older women to stand, appears to be another strong determinant of sedentary time. Most participants complained of a lack of facilities and opportunities that encourage or enable older women to be active and regretted that they were mostly offered activities that required them to be seated. This might be the result of a widespread risk adverse culture within organizations and policies which cater for older women [31]. 

Weather conditions and urban design were also emerging themes which fall within the environmental category of determinants. In Scotland, were the study took place, poor weather was described by the participants as a reason to sit more than they would wish to and lead to them feeling less motivated to be active. Counter intuitively, the participants blamed a lack of sitting facilities spaced around the urban environment or within community facilities as a reason to stay sedentary. They explained that more seats would enable them to rest when needed and pace themselves, giving them increased confidence which in turn would allow them to venture further outside and do more standing up. This is not an issue that has been considered by research on neighborhood walkability [44] or built environment design to promote self-efficacy and physical activity [45].

There was not a strong interest in reducing the amount of sitting time amongst this sample of older women. Fear of disrupting daily routines built around managing already difficult circumstances, a lack of energy and a sense that they already did the best they could were the most strongly expressed reservations. However there was a lot of interest in changing their pattern of sitting and a genuine hunger for engaging in more standing activities, provided they can rest and pace themselves when needed. 

The results of this qualitative investigation suggest that there are some opportunities and factors to be considered in the development of interventions.

The pattern of sedentary time throughout the day could determine the effectiveness of interventions. Morning interventions could be more successful but would target the least sedentary part of the day therefore yielding a limited change. Most participants reported thinking that morning intervention would be easier for them. However some of them recognized that they would welcome the chance to change their pattern of behavior. It is therefore possible that interventions which target sedentary times during the afternoon and evening might be more successful and effective in reducing total sitting time. This might however require spreading the interruption of sitting periods throughout the day, using strategies such as pacing. Frequent short interruptions while sitting was thought by some of the participants as the easiest and most manageable way for them to change their sedentary behavior. This implies that intervention monitoring should focus not only on the achieved reduction of sedentary behavior but also in measuring a change in pattern.

Interventions should pay attention to the individual pattern of sedentary behavior and not disrupt periods of sitting that are used as coping strategies or deemed beneficial by older women. Therefore it would be sensible to work with older women on an individual basis to both monitor the pattern of sitting and identify those beneficial periods. This will require a system of classification of sedentary periods to be used [46].

Short term benefits, such as relieving and managing stiffness, pain or depression, seem to be strong motivating factors in breaking long periods of sitting that are already adopted spontaneously by older women. Interventions should consider harnessing these salutogenic behavior adaptations and the person’s own coping strategies to foster sustainable change. 

Together these last two points suggest that interventions should be individualized and tailored [47] and might explain the success in the short term reduction of sedentary behavior obtained by Gardiner *et al*. [16].

The desire for social interaction and for assuming a purposeful role within society, such as caring for others, appears to be other opportunities for encouraging behavioral change. This seems to advocate the delivery of interventions through peer-mentoring schemes which have demonstrated success in the areas of fall prevention and physical activity promotion [48,49].

Finally, the activities which community services offer older women appear to lack the stimulation which encourages them to stand up. Interventions should also consider targeting organizations which provide services to older people. This might require changing organizational culture and staff training as well as looking at the design of the interior and urban environment to ensure there are enough resting spaces in the right location and density.

It is not possible to tell from this study if similar factors determine sedentary behavior in older men. However, there was no indication in the data that the factors reported by the women in this study are gender specific. The participants tended to talk about older people and the effect of ageing rather than making statements about older women, yet they answered from women perspectives. It is reasonable to assume that a number of these factors, in particular physical factors, might determine older men sedentary behavior, but it is likely their effect is of a different magnitude and nature. It is also conceivable that older men might express different view and qualitative investigations should be undertaken to fill this research gap.

There are some limitations to this study. Firstly, the sample was small, even for a qualitative study, although it fulfilled the criteria for saturation according to Morse [50]. The sample was fairly homogeneous so this limits the ability to generalize the results. Finally, the themes extracted could not be triangulated with the screening questionnaire (Table 1).

## 5. Conclusions

Despite these few limitations, this study has identified, for the first time, that the perceptions which older adults have of sedentary behavior and its determinants are very specific. Not only are they different from those relating to physical activity, but they are not described in detail in any current model of sedentary behavior. The results of this study can inform the development of interventions to reduce sedentary time and the associated health and economic burden that it causes.
